# Gender in mental health: Relationship of spirituality, social support, and COVID-19-related fear among heterosexual and LGBTQ+ youth

**DOI:** 10.3389/fsoc.2022.1102664

**Published:** 2023-01-04

**Authors:** Rowalt Alibudbud

**Affiliations:** Department of Sociology and Behavioral Sciences, De La Salle University, Manila, Philippines

**Keywords:** COVID-19, Philippines, social support, spirituality, mental health, gender, LGBT persons, sexual and gender minorities

## Abstract

**Introduction:**

The youth is a vulnerable population to COVID-19-related fear. Among them, those with LGBTQ+ identities are at higher risk. Given the posited protective effects of spirituality and social support on fear, this study explored their effects on COVID-19-related fear among heterosexual and LGBTQ+ youth.

**Materials and methods:**

This cross-sectional study recruited 137 respondents Filipinos aged 18–30 years old. The respondents answered a survey containing a sociodemographic questionnaire, 12-items Multidimensional Scale of Perceived Social Support (MSPSS), 20-items Core Dimensions of Spirituality Questionnaire (CDSQ), and 7-items Fear of COVID-19 Scale (FCS). After, the data were analyzed using means, frequencies, Mann-Whitney *U*-tests, and correlation coefficients.

**Results:**

Social support from friends was negatively correlated with COVID-19-related fear among heterosexual respondents, R = −0.219, *p* = 0.029. Search for meaning positively correlated with COVID-19-related fear among LGBTQ+ respondents, R = 0.395, *p* = 0.016. Heterosexual respondents also have a higher belief in God (*U* = 1,004, *p* < 0.001) and feelings of security (*U* = 1,110.5, *p* < 0.001) than their LGBTQ+ counterparts.

**Discussion:**

These findings suggest that social support from friends is protective against COVID-19-related fear among heterosexual youth but not among LGBTQ+. In addition, a high search for meaning can heighten COVID-19-related fear among LGBTQ+. Finally, these findings can be explained by the higher rates of discrimination against LGBTQ+ than heterosexual youth. Thus, gender-responsive mental healthcare is needed to address the youth's fears as society recovers from the pandemic.

## 1. Introduction

The COVID-19 pandemic has caused significant anxiety and fear among Filipinos, with the youth at higher risk than other age groups (Tee et al., 2020). Similar to other countries in Southeast Asia, LGBTQ+ Filipinos have also shown greater vulnerability to ill mental health than their peers (Alibudbud, 2021). Their higher rates of mental health problems have been attributed to the high rates of discrimination against them (Tan and Saw, 2022).

Previous studies among Filipinos found that fear and anxiety can be mitigated by providing adequate health information and economic support and lessening quarantine and lockdown periods (Tee et al., 2020). However, factors stemming from Filipino cultural features have yet to be compared (Tee et al., 2020). Among others, Filipino culture has closely knitted communities that provide social support. Among the youth, this is manifested in closely bonded peer support groups called “barkadahan” (Alibudbud, 2021). Filipino culture also emphasizes spirituality and religion based on Roman Catholicism (Lagman et al., 2014; Alibudbud, 2021). This high spirituality among populations is protective against fear and anxiety (Morgan and Bhugra, 2010). However, LGBTQ+ Filipinos are reportedly marginalized from this Filipino cultural practice that may be protective of heightened fear and anxiety (UNDP USAID., 2014; Human Rights Watch, 2017; Alibudbud, 2021). Thus, they may not have the same protection from these cultural features compared to their peers.

Given the heightened COVID-19-related fear among the youth and the higher vulnerability of those LGBTQ+ identities, social support and spirituality can be explored and compared as possible determinants of COVID-19-related fear among Filipino heterosexual and LGBTQ+ youth.

## 2. Materials and methods

This cross-sectional study determined the relationship between social support, spirituality, and COVID-19-related fear among heterosexual and LGBTQ+ youth. This study was a component of a larger study that investigated the relationship between COVID-19-related fear and election participation among young Filipino adults. Local ethical approval and informed consent were secured before data collection. Likewise, numerical codes were used in lieu of identifying data.

The sample size for this study was computed using G^*^power 3.1, a statistical power analysis program (Faul et al., 2007). The present study utilized the suggested parameters for sample size computation by Cohen (1988) and Lakens (2013), including a moderate effect size of 0.5, an alpha error of 0.05, and a power of 0.8. The minimum total sample size needed was 132. This study included individuals with Filipino citizenship and an age range of 18–30 years old. Filipino citizens aged 17 or lower and 31 or higher were excluded from this study. To avoid the health risks during the COVID-19 pandemic, the study opted to invite and recruit potential respondents through convenience sampling in online mediums, such as social media and public forum platforms. The data collection lasted for 2 months. After the data collection period had elapsed, a community sample of 137 respondents was recruited.

Before answering the survey questionnaire, the respondents indicated their informed consent. The survey contained several sections, including a sociodemographic questionnaire, 12-items Multidimensional Scale of Perceived Social Support (MSPSS), 20-items Core Dimensions of Spirituality Questionnaire (CDSQ), and 7-items Fear of COVID-19 Scale (FCS). The MSPSS, CDSQ, and FCS measured the respondents' social support, spirituality, and COVID-19-related fear. These questionnaires have previously confirmed validity and reliability (Zimet et al., 1990; Hardt et al., 2012; Ahorsu et al., 2022). For this study, the MSPSS, CDSQ, and FCS showed acceptable internal consistency with a Cronbach's alpha of 0.847, 0.883, and 0.866, respectively.

The data were described using means and frequencies and analyzed using Mann-Whitney U-tests to determine the significant differences between spirituality and social support among heterosexual and LGBTQ+ respondents. In addition, the relationship between spirituality and social support with COVID-19-related fear was analyzed using correlation coefficients. A *p*-value of <0.05 was considered significant. All statistical tests were analyzed using SPSS.

## 3. Results

The average age of the respondents was 20.06 (SD = 2.90). The majority of the respondents were females (*n* = 90, 65.70%), had heterosexual orientation (*n* = 100, 73.00%), were high school graduates (*n* = 105, 76.6%), single (*n* = 135, 98.54%), Catholic (*n* = 104, 75.90%), and have a monthly household income of less than PHP 10,957 per month (*n* = 80, 58.40%). This income level indicates that most respondents came from the lowest income class.

Table 1 shows that social support from friends was negatively correlated with COVID-19-related fear among heterosexual respondents, R = −0.219, *p* = 0.029. Contrastingly, the search for meaning positively correlated with COVID-19-related fear among LGBTQ+ respondents, R = 0.395, *p* = 0.016. These findings suggest that social support from friends may be a protective factor for COVID-19-related fear among heterosexual respondents, while a high search for meaning may be a risk factor for COVID-19-related fear among LGBTQ+ respondents.

**Table 1:** Differences and correlation of social support and spirituality with fear of COVID-19 among heterosexual and LGBTQ+ respondents (*n* = 137).

	**Descriptive statistics**						**Correlation coefficient**			
	**Heterosexual**		**LGBTQ**+		**Mann Whitney** ***U*** **tests**		**Heterosexual**		**LGBTQ**+	
	**Mean**	**SD**	**Mean**	**SD**	***U***	***p***	***R***	***p***	***R***	***p***
**Social support**										
Special someone	20.47	6.59	19.30	7.60	1,710	0.496	−0.164	0.102	0.163	0.335
Family	21.05	4.94	19.19	4.85	1,407	0.031	−0.026	0.799	0.187	0.268
Friend	23.57	3.89	23.84	4.29	1,699.5	0.462	−0.219^*^	0.029	−0.013	0.938
**Spirituality**										
Belief in god	19.84	4.56	14.73	6.59	1,004^*^	< 0.001	−0.082	0.415	0.215	0.201
Search for meaning	20.09	3.08	19.92	3.65	1,848.5	0.994	0.066	0.515	0.395^*^	0.016
Mindfulness	21.44	2.65	21.86	2.03	1,749	0.621	−0.061	0.545	0.198	0.241
Feeling of security	15.76	4.01	12.95	4.24	1,110.5^*^	< 0.001	0.024	0.812	0.052	0.759

* *p* < 0.05.

Table 1 also shows that heterosexual respondents have a higher belief in God (U = 1,004, *p* < 0.001) and feelings of security (U = 1,110.5, *p* < 0.001) than their LGBTQ+ counterparts.

## 4. Discussion

This study found that social support from friends may be a protective factor against COVID-19-related fear among heterosexual respondents but not among LGBTQ+ respondents. Sexual and gender identity concealment can explain this lack of protective effects among LGBTQ+ since they may have higher worries about gender-based discrimination (UNDP USAID., 2014; Human Rights Watch, 2017; Alibudbud, 2021). Thus, the effect of social support may be lower in mitigating fear among LGBTQ+ since they may be unable to physically and overtly support their peers with COVID-19-related fear due to discrimination if identified as a person with an LGBTQ+ identity (UNDP USAID., 2014; Human Rights Watch, 2017; Alibudbud, 2021).

It was also found that a high search for meaning can be a risk factor for COVID-19-related fear among LGBTQ+. This finding can be explained by the gender-based discrimination they received in their families and homes (UNDP USAID., 2014; Human Rights Watch, 2017). Since their homes are not safe for exploring their sexual and gender identities, they may seek to understand their identities in their outside environment where they have higher exposure to COVID-19, resulting in greater concerns and fear of contracting COVID-19.

Notably, the study also shows that Belief In God and Feelings of Security were lower among LGBTQ+ youth than their heterosexual peers. The lower feelings of security can be due to the higher rates of discrimination against them that may cause them to feel unsafe in their environment. These discriminations against LGBTQ+ youth can be based on traditional religious beliefs (UNDP USAID., 2014; Human Rights Watch, 2017; Manalastas et al., 2017; Alibudbud, 2021). This use of religion as a ground for discrimination may explain their lower belief in God.

While this study showed that the effect of social support and spirituality on COVID-19 fear varied based on gender, further studies are recommended. For example, future research can employ non-purposive sampling designs and recruit a larger sample size to improve the study's generalizability and power. In addition, qualitative methods can explore the mechanisms and meanings of social support and spirituality related to mental health among people of different genders and sexuality. Nonetheless, the study provides evidence that gender-responsive mental healthcare beyond traditional binary models of men and women may be needed to equitably address the youth's COVID-19-related fears as society recovers from the pandemic. As a start, the gender-related attitudes of mental healthcare and other individuals highly involved in youth interactions (i.e., schools and workplaces) can be improved. In doing so, gender awareness, sensitivity, and training programs for mental health professionals, school staff, and workplace managers can be developed and expanded to improve gender-related attitudes (Woodford et al., 2012; Alibudbud, 2021; Okanlawon, 2021).

## Data availability statement

The data analyzed in this study is subject to the following licenses/restrictions: The datasets for this study is available upon request and permission from Jose Mari Gabriel Tumanan, Miranda Monserrat Bardos, Archibald Noel Po, and Kurt Travis Arbolante. Requests to access these datasets should be directed to rowalt.alibudbud@dlsu.edu.ph.

## Ethics statement

The studies involving human participants were reviewed and approved by De La Salle University Integrated School. The patients/participants provided their written informed consent to participate in this study.

## Author contributions

RA had substantial contributions to the design, drafting, revision, acquisition, interpretation, and final approval of the data and work.
